# A case report of liver abscesses caused by *Fusobacterium necrophorum* in immunocompetent patient and review of the literature

**DOI:** 10.1016/j.heliyon.2023.e18535

**Published:** 2023-07-21

**Authors:** Xiaoqiang Zhang, Jiyun Zhou, Yinhong Zhu, Xufang Ye, Dehua Bo, Tianjiao Tang, Xiaojing Huang, Tingting Qu

**Affiliations:** a Department of Infectious Disease, First People's Hospital of Linping District, Hangzhou, China; b Department of Laboratory Medicine, First People's Hospital of Linping District, Hangzhou, China; c Department of Ultrasonography, First People's Hospital of Linping District, Hangzhou, China; d Department of Infectious Disease, Liangzhu Hospital, Yuhang District, Hangzhou, China; e State Key Laboratory for Diagnosis and Treatment of Infectious Diseases, National Clinical Research Center for Infectious Diseases, Collaborative Innovation Center for Diagnosis and Treatment of Infectious Diseases, The First Affiliated Hospital, Zhejiang University School of Medicine, Hangzhou, China

**Keywords:** *Fusobacterium necrophorum*, Liver abscess, Immunocompetence, Literature review, Case report

## Abstract

**Background:**

*Fusobacterium necrophorum* is an anaerobic Gram-negative bacterium that can lead to opportunistic infections, including Lemierre's syndrome and less common presentations of metastatic diseases. However, liver abscesses infected by *Fusobacterium necrophorum* in clinical settings are rarely reported, particularly in people with normal immune function.

**Case presentation:**

A 35-year-old Chinese man was admitted with hyperthermia and abdominal pain that had persisted for three days. The patient continued to have a fever with a maximum temperature of 39.8 °C during hospitalization. Computed Tomography revealed multiple low-density lesions in the liver, which were diagnosed as liver abscesses caused by *Fusobacterium necrophorum* infection through blood culture and anaerobic liver abscess fluid culture. After simple local percutaneous abscess drainage and effective anti-infective therapy, the patient achieved complete remission.

**Conclusions:**

Results of our literature search query revealed rare reports of liver abscesses infected by *Fusobacterium necrophorum*. We recommend that *Fusobacterium necrophorum* infection be considered in diagnosis special situations of liver abscess.

## 1 Background

*Fusobacterium necrophorum* is an anaerobic Gram-negative bacterium that can cause Lemierre's syndrome, a disease characterized by post-anginal infection followed by internal jugular vein thrombophlebitis. It can also lead to metastatic diseases, including liver abscesses, renal abscesses, endocarditis, brain abscesses, and soft tissue abscesses [[1], [2], [3]]. In 1936 André Lemierre first described post-anginal infection in adolescents and young adults, which had a mortality rate of 90%. However, since the widespread use of antibiotics, the morbidity and mortality of diseases associated with *Fusobacterium necrophorum* have significantly decreased [4,5]. Today, the mortality rate is unusual, at 5–9% [6]. However, due to the rarity of this bacterium and difficulties in culture, it may be misdiagnosed in the clinic [7,8]. In this report, we present a case of multiple liver abscesses caused by *Fusobacterium necrophorum* without Lemierre's syndrome or other metastatic diseases. Furthermore, we conducted a search of the PubMed database and found few documented associations between Lemierre's syndrome and metastatic diseases, particularly liver abscesses infected by *Fusobacterium necrophorum*. This paper aims to improve recognition of the disease by reporting on the diagnosis and treatment of multiple liver abscesses associated with *Fusobacterium necrophorum* infection.

## 2 Case presentation

A 35-year-old Chinese man who had a one-year history of wisdom teeth and no previous medical history was admitted to the hospital on April 14, 2020, with hyperthermia and abdominal pain that had been present for three days. The patient reported being exposed to rain and feeling cold three days before the onset of symptoms. Upon admission, the patient presented with hyperthermia, chills, mild cough, expectoration, headache, nausea, and watery stools (4–5 times per day). He was initially evaluated at The First People's Hospital of Linping District, Hangzhou, China, where laboratory results on April 14 showed a white blood cell count of 21.6 × 10^9^/L with a neutrophil ratio of 93.4% and a C-reaction protein (CRP) level of over 200mg/L. Abdominal computed tomography (CT) showed multiple low-density shadows with unclear boundaries in the liver, prompting immediate admission to our hospital.

**Fig. 1:**
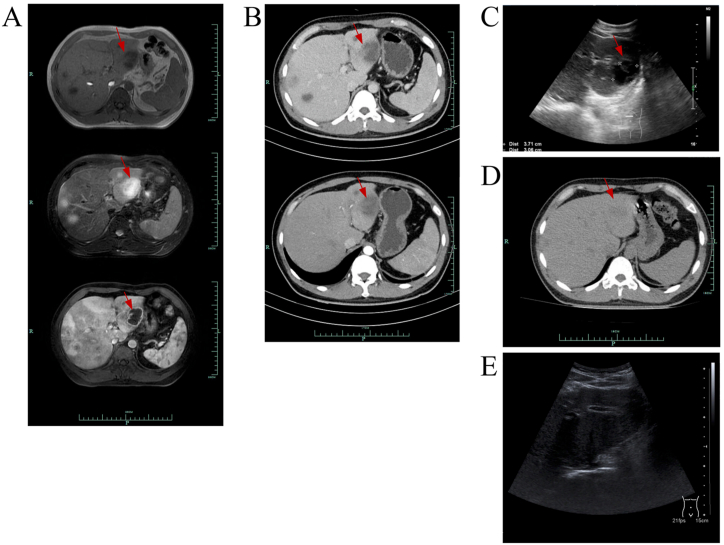
A. Abdominal enhance MRI shows multifocal hypointense signals in T1, hyperintense signals in T2, and obvious enhancement in DWI, and the largest lesion is located in the Ⅲ segment of the liver, size to 3.2 × 4.2cm. The red arrow means the lesion site. B. Abdominal Contrast-enhanced CT indicates multiple low-density lesions in the liver, and the significant abscesses size to 3.7 × 4.2cm. C. Abdominal ultrasonography shows multiple low-density shadows in the liver, and the significant abscesses size to 3.71 × 3.06cm. D. The post-treatment check CT shows that the maximum diameter of liver abscesses was 1.5 × 1.6cm. E. The post-treatment check abdominal ultrasonography shows no abscess. (For interpretation of the references to colour in this figure legend, the reader is referred to the Web version of this article.)

On the day of admission, the patient presented with a body temperature of 38.5 °C, heart rate of 117 beats per minute, respiratory rate of 18 breaths per minute, and blood pressure of 117/65 mmHg. A comprehensive physical examination was conducted, which did not reveal any abnormalities except for the aforementioned vital signs. Cefoperazone/sulbactam was administered intravenously at a dose of 2g every 8 hours, and blood cultures for aerobic and anaerobic bacteria were obtained before initiating anti-infective therapy. However, laboratory results obtained after hospital admission (see Table 1) showed elevated levels of white blood cells, with a neutrophil ratio of 85%, and a CRP level exceeding 200mg/L. Additionally, the patient's alanine aminotransferase (ALT) was 47 U/L, aspartate aminotransferase (AST) was 47.04U/L, total bilirubin was 16.8L, direct bilirubin was 9.0/dL, alkaline phosphatase (ALP) was 88.0/L, γ-glutamyltransferase (γ-GT) was 36.0U/L, and procalcitonin (PCT) was 68.54ng/ml. A contrast-enhanced CT of the abdomen revealed the presence of low-density shadows in the liver, with the largest measuring 3.7*4.2cm and located in the left lobe of the liver, suggestive of liver abscesses. The enhanced magnetic resonance imaging (MRI) showed multifocal hypointense signals in T1, hyperintense signals in T2, and significant enhancement in Diffusion-Weighted Magnetic Resonance Imaging (DWI) (see Fig. 1A and B). And the liver was diffusely hypoechogenic on ultrasonography (Fig. 1C).

**Table 1:** Results of blood tests.

Blood Tests	2nd day after admission	4th day after admission	11th day after admission	17th day after admission	6th day after discharge	27th day after discharge
WBC, × 10^9^ cells/L	22.6	17.2	14.0	8.8	10.1	8.1
Neutrophilpercentage, %	85.0	77.8	75.2	58.3	58.0	50.3
Hb, g/L	155	136	133	135	144	145
PLT, 10^9^ cells/L	78	129	521	485	344	269
CRP, mg/L	>200	133.3	41.5	5.8	2.2	<0.50
PCT, ng/ml	68.54	8.98	2.06	0.32	<0.05	/
TB, μg/L	16.8	/	10.9	5.70	12.10	16.40
DBil, μg/L	9.0	/	3.60	2.70	3.2	2.50
ALB, g/L	33.3	26.6	27.7	32.3	38.7	46.2
ALT, U/L	47.0	54	97.0	88.0	61.0	46.0
AST, U/L	47.0	/	41.0	46.0	43.0	29.0
γ-GT, U/L	36.0	/	80.0	93.0	96.0	67.0
ALP, U/L	88.0	/	111.0	124.0	113.0	74.0
Anaerobic Blood Culture			Positive			
Aerobic Blood Culture			Negative			

Note: “/” in the table means that the data was missing.Abbreviations: WBC white blood cell, Hb hemoglobin, PLT platelet, TB total bilirubin, DBil direct bilirubin; ALB albumin.

**Fig. 2:**
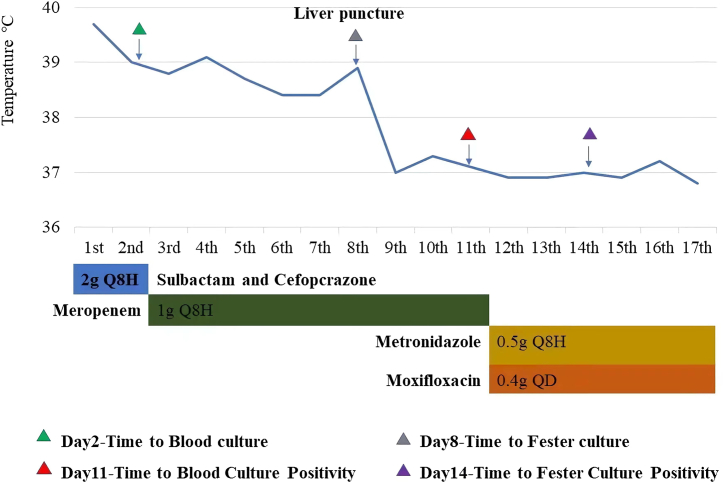
Schematic diagram of the patient's clinical course.

**Fig. 3:**
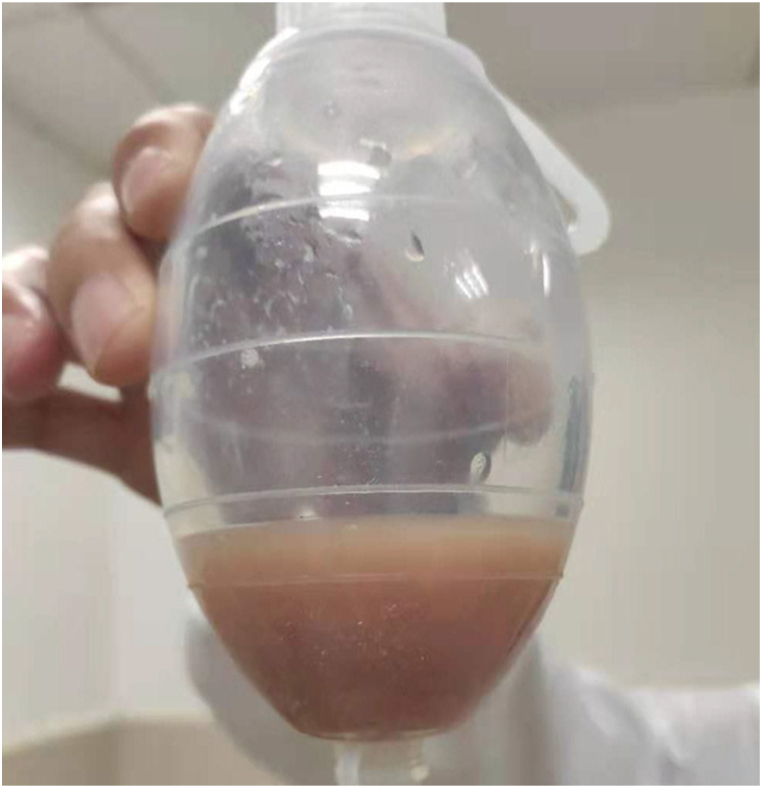
The percutaneous transhepatic abscesses drainage.

**Fig. 4:**
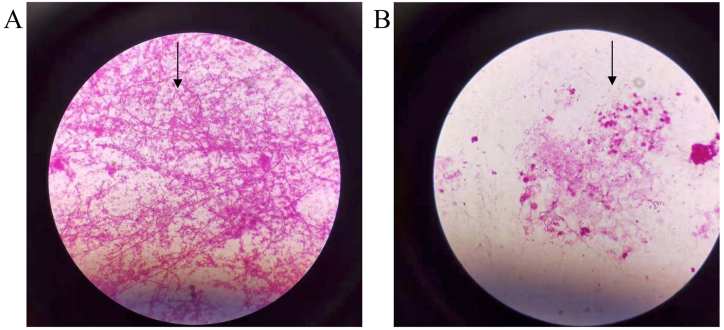
**A.** Using Gram staining, the *Fusobacterium necrophorum* was evaluated in a light microscope (1000x)-the samples originating from anaerobic blood culture bottle. **B.** Using Gram staining, the *Fusobacterium necrophorum* was evaluated in a light microscope (1000x)-the samples originating from fluid of liver abscesses.

Moreover, the patient continued to have a sustained fever, and since the initial treatment showed poor efficacy, we changed the antibiotic to Meropenem 1g intravenously every 24 hours (refer to Fig. 2). On the eighth day after admission, percutaneous transhepatic abscess drainage was performed (refer to Fig. 3), and the fluid from paracentesis of the liver abscesses was immediately sent for aerobic and anaerobic blood culture showed positive results while the aerobic culture was bacteria immediately. On the eleventh day after admission, the anaerobic blood culture showed positive results while the aerobic culture was negative (refer to Fig. 4A). The bacteria from the positive anaerobic culture were identified as *Fusobacterium necrophorum* through MALDI-TOF mass spectrometry, and the Gram-staining of *Fusobacterium necrophorum* is shown in Fig. 2A. Subsequently, the patient was diagnosed with multiple liver abscesses infected by *Fusobacterium necrophorum*, and the antibiotic therapy program was promptly changed from Meropenem to a combination of Metronidazole 0.5g and Moxifloxacin 0.4g intravenously per day. The same result was obtained in the fluid culture of liver abscesses (refer to Fig. 4B) on the 14th day after admission, and the white blood cell count returned to normal on the 17th day. After 17 days of hospitalization, the patient fully recovered and was discharged (refer to Fig. 2).

After discharge, the patient continued oral anti-infection treatment with Metronidazole 0.6g three times per day combined with Moxifloxacin 0.4g per day for 2 weeks. A follow-up CT scan on May 6th showed that the maximum diameter of the liver abscesses has decreased to 1.5 × 1.6cm (Fig. 1D). Additionally, abdominal ultrasonography conducted on May 27th indicated that the lesions were significantly smaller, with a maximum diameter of approximately 1.68 × 1.42cm (Fig. 1E), and blood tests showed normal results. The patient did not return for re-examination after May 27th. Telephone follow-ups were conducted after 3 months, and no relapse was reported. In this case report, the possibility of hospital infection and the relationship between the infection and the use of antibiotics in the early stages were not considered because a blood culture had been taken on the second day after admission; and it showed anaerobic positivity.

## 3 Discussion and conclusions

As we know, the liver is the confluence of the portal and systemic circulations, and its peculiar anatomy may lead to the development of liver abscesses, which are the most common type of visceral abscesses. Furthermore, liver abscesses are more easily developed in immunocompromised patients, such as those with tumors, diabetes, and immune diseases [9]. Severe infections caused by bacteria or histolytic amoeba, such as *Klebsiella pneumoniae*, are a common source of portal vein infections [10]. However, it is rare for liver abscesses to be infected by *Fusobacterium necrophorum* in a clinical setting, particularly in people with normal immune function. *Fusobacterium necrophorum* is a strictly anaerobic organism that is difficult to isolate from culture and is rarely reported. Thus, infections associated with *Fusobacterium necrophorum* may be missed in the clinic.

*Fusobacterium necrophorum* is an anaerobic gram-negative bacterium that can produce pathogenic agents, including endotoxin, leukotoxin, platelet factor, leukotoxin, and hemolysin. This bacterium is usually less resistant to physicochemical factors such as potassium permanganate, formaldehyde solution, high temperature, and intense sun exposure. It can survive for approximately 10–30 days in soil and 50 days in excrement. *Fusobacterium necrophorum* is a zoonosis that can lead to foot rot, swollen joints, and liver abscesses [11]. It can adhere to the oral cavity, genitourinary tract, and gastrointestinal tract in the human body [12]. When the immune function of the body is abnormal, *Fusobacterium necrophorum* may be able to cause opportunistic infections through the oropharynx to the internal jugular vein thrombophlebitis, leading to Lemierre's syndrome or migratory infections, such as infective endocarditis, brain abscesses, pleurisy, and liver abscesses [[13], [14], [15]].

We conducted a literature search on PubMed using the keywords “*Fusobacterium necrophorum*” AND “liver abscess”, and excluded non-English language and non-human case reports. Our search yielded 57 cases, out of which only 20 cases involved immunocompetent patients. The details of these 20 cases, including the sources of infection, metastatic lesions, antibiotics therapy, and outcomes, have been summarized in Table 2. Four cases had a significant history of dental and periodontal disease, six cases had respiratory tract infections, four cases had digestive tract infections, one case had appendicitis, one case had a history of the gynecological procedure, and one case had an intrauterine device insertion. Our patient had a history of periodontal disease for one year, leading us to consider the oral cavity as the source of infection. All 20 patients survived, possibly due to prompt etiological diagnosis and therapy. However, it is important to note that all patients in our analysis were immunocompetent, and immune diseases may require more vigilance. Clinicians should not overlook the possibility of *Fusobacterium necrophorum* infection in patients with symptoms such as fever, chills, nausea, abdominal pain, diarrhea, and a history of dental and periodontal disease. The main treatments for liver abscesses are drainage and effective anti-infective therapy. In the early course of our patient's illness, an ultrasound examination indicated that the abscess was not liquefied and presented with multiple abscesses. Therefore, puncture treatment was not performed initially, which may have contributed to the patient's lack of improvement despite receiving powerful antibiotics continuously.

**Table 2:** Summary of Case Reports of *Fusobacterium necrophorum* liver abscess among immunocompetent patients.

Number/References	Age/Gender	Possible infection source	Number of liver abscesses	LS	Oher metastatic lesion	Drainage	Antibiotics given	Outcome reported in the cited literature
1. [5]	44/male	upper respiratory tract infection	multiple	yes	no	no	-Oral Cefdinir (1 week) -IV Doripenem (9 days) -Oral Levofloxacin (agent period time not specified)	Alive
2. [7]	65/male	digestive tract infection	multiple	no	no	yes	-Ciprofloxacin and metronidazole (1 week) -Oral Ceftriaxone 1g daily and Metronidazole 500 mg three times daily (agent period time not specified)	Alive
3. [13]	41/male	unknown	multiple	no	Hepatic vein pylebitis, Brain abscesses	yes	-Vancomycin 1g, Ceftriaxone 2g, Metronidazole 500mg (agent period time not specified)	Alive
4. [9]	30/male	digestive tract infection	multiple	no	no	yes	-Metronidazole and Piperacillin/tazobactam (agent -time period not specified) -Oral Levofloxacin 750mg, Metronidazole 500mg (agent -time period not specified)	Alive
5. [16]	49/male	respiratory tract infection	single	no	Endocarditis	yes	-IV Piperacillin/tazobactam -IV Ampicillin/sulbactam (6 weeks) -IV Ertapenem (agent -time period not specified)	Alive
6. [17]	27/male	Unknown	single	no	no	yes	-Ampicillin, Gentamicin, Metronidazole (agent -time period not specified)	Alive
7. [18]	18/male	upper respiratory tract infection	multiple	no	no	yes	-Unidentified	Alive
8. [19]	88/female	digestive tract infection	multiple	no	no	yes	-Ceftriaxone and Metronidazole (>10days)	Alive
9. [20]	51/female	digestive tract infection	multiple	no	no	yes	-Ceftriaxone and Metronidazole (9 days) -IV Ertapenem (2 weeks) -Oral Amoxicillin/clavulanate (4 weeks)	Alive
10. [21]	57/male	Dental cleaning and examination	single	no	no	yes	-Metronidazole and Piperacillin/tazobactam (unknown)	Alive
11. [22]	19/female	History of gynecological procedure	multiple	yes	no	yes	-Amoxycillin (1 week) -Metronidazole, Benzylpenicillin, Ciprofloxacin (6.5week)	Alive
12. [23]	28/male	upper respiratory tract infection; history of a dental examination	multiple	yes	Sepic pulmonary emboli	no	-Ceftriaxone (6 days) -Meropenem (14days)	Alive
13. [24]	22/male	unknown	multiple	no	no	yes	-Gentamicin, Clarithromycin, Metronidazole, Ceftriaxone (6 weeks)	Alive
14. [25]	24/male	Dental and periodontal disease	multiple	yes	Brain abscesses	no	-IV Ceftriaxone, Vancomycin, Metronidazole (agent period time not specified)	Alive
15. [26]	51/female	Dental and periodontal disease	multiple	no	Endocarditis	no	-IV Meropenem (antibiotics were discontinued for drug eruption) -IV Ceftriaxone (12 days) -IV Ceftriaxone with IV Metronidazole (4 weeks)	Alive
16. [27]	19/male	Appendicitis	single	no	no	yes	Unidentified	Alive
17. [28]	25/male	upper respiratory tract infection and digestive tract infection	single	no	Endocarditis, Splenic abscesses	yes	-IV Piperacillin/tazobactam 3.375g per 6 hours (3 weeks) -IV penicillin G 24million units per day (6 weeks)	Alive
18. [29]	36/male	Dental and periodontal disease	multiple	no	No	yes	-Ceftriaxone 2g daily (agent period time not specified) -Ampicillin/sulbactam 12g daily (agent period time not specified)	Alive
19. [30]	29/female	Intrauterine device insertion	multiple	yes	Sepic pulmonary emboli	no	-Unidentified	Alive
20. [31]	21/male	upper respiratory tract infection	multiple	no	no	yes	-IV Piperacillin/tazobactam, Metronidazole (agent period time not specified)	Alive

Abbreviation: LS Lemierre's syndrome.

The definitive etiological diagnosis of liver abscesses is usually based on bacterial culture. On the eleventh day of the patient's hospitalization, anaerobic cultures of the blood grew *Fusobacterium necrophorum*, and on the third day after that, the same results were displayed in the liver abscess fluid (Fig. 4B). Upon reviewing the available literature, we found that five of the 20 cases in the literature review presented with Lemierre's syndrome. In consideration of this possibility, we examined the neck vascular ultrasound, which revealed no abnormalities. Additionally, to rule out infection of other organs, we conducted relevant examinations such as brain MRI, brain CT, thoracic CT, cardiac ultrasound, and abdominal ultrasound, all of which yielded negative results. Thus, we excluded the possibility of Lemierre's syndrome and other metastatic diseases.

The treatments of liver abscesses are relatively time-consuming, and the main treatments are drainage with effective anti-infective therapy. Therefore, abscess drainage was performed under ultrasound guidance. Equally important was effective anti-infective therapy [9]. Through a literature search, we discovered that most broad-spectrum antibiotics used in sepsis coverage are sensitive to *Fusobacterium necrophorum*, including ampicillin, piperacillin, ceftriaxone, cefoperazone/sulbactam, imipenem, meropenem, levofloxacin, and metronidazole [5] (treatments of 20 cases were summarized in Table 2). However, *Fusobacterium necrophorum* was found to be resistant to teicoplanin and vancomycin. Nonetheless, bata-lactamase activity exists in this bacterium [16]. Based on this, we chose to administer Metronidazole 0.5g combined with moxifloxacin 0.4g intravenously per day. Following discharge, the patient was instructed to continue oral moxifloxacin treatment for two weeks and has had no relapses since.

In this case, effective anti-infective therapy was crucial for a good prognosis, highlighting the importance of prompt and accurate diagnosis. The possibility of *Fusobacterium necrophorum* infection should be considered in patients with liver abscesses of unknown cause, and anaerobic cultures of blood and liver abscess should be performed to detect this bacterium. In cases where cultures are negative, metagenomic next-generation sequencing (mNGS) can be used to determine the etiology. especially when the cultures were negative.

## Declarations

### Ethics approval and consent to participate

This study has been reviewed and approved by the ethical research committee of the First People's Hospital of Linping District, Hangzhou (approval No.2021013). Informed consent was obtained from the patient for publication of their clinical data.

### Consent to publish

Written informed consent was obtained from the patient for publication of this case report and any accompanying images. A copy of the written consent is available for review by the Editor of this journal.

### Author contribution statement

All authors listed have significantly contributed to the investigation, development and writing of this article.

### Data availability statement

Data included in article/supp. material/referenced in article.

## Declaration of competing interest

The authors declare that they have no known competing financial interests or personal relationships that could have appeared to influence the work reported in this paper

## List of abbreviations

**CRP**: C-reaction protein
**CT**: Computed Tomograph
**ALT**: alanine aminotransferase
**AST**: aspartate aminotransferase
**ALP**: alkaline phosphatase
**γ-GT**: γ-glutamyltransferase
**PCT**: procalcitonin
**MRI**: Magnetic Resonance Imaging
**mNGS**: Metagenomic next-generation sequencing
